# Rectal Metastasis Causing Rectal Constriction From High-Grade Urothelial Carcinoma

**DOI:** 10.7759/cureus.79416

**Published:** 2025-02-21

**Authors:** Hassan Chamas, Brian Hoffmann, Jonathan Millard

**Affiliations:** 1 Medicine, Edward Via College of Osteopathic Medicine, Blacksburg, USA; 2 General and Colorectal Surgery, Carilion Clinic, Christiansburg, USA; 3 Anatomy, Edward Via College of Osteopathic Medicine, Blacksburg, USA

**Keywords:** abdominal imaging, high grade urothelial carcinoma, pelvic imaging, rectal constriction, rectal endoscopic ultrasound, rectal metastasis, structural constipation, upper urinary tract obstruction

## Abstract

Urothelial carcinoma arises from the urothelial cells that line the urinary tract, which extends from the renal pelvis to the urethra. Bladder cancer commonly metastasizes to the lymph nodes, bones, liver, and lungs. In rare cases, such as in this case report, it can metastasize to the rectum. In this case report, a 62-year-old male patient presented initially with constipation, anal discharge, perineal pain, mild difficulty urinating, and a rectal mass. The first CT abdomen and pelvis with IV contrast revealed the rectum, sigmoid colon, and proximal descending colon to have circumferential wall thickening with retroperitoneal enlarged lymph nodes. An MRI pelvis with and without contrast revealed marked concentric mass-like wall thickening of the entire rectum, with extensive mesorectal and pelvic lymphadenopathy. A second CT pelvis with IV contrast again revealed extensive rectal wall thickening and irregular thickening of the bladder wall. Rectal endoscopic ultrasound (EUS) with biopsy was performed and found the rectal mass to be high-grade metastatic urothelial carcinoma. Transurethral resection of bladder tumor (TURBT) found high-grade urothelial carcinoma involving the bladder trigone. This case provides a unique observation of a patient whose presenting symptoms were due to metastatic disease and who did not begin suffering major urinary symptoms suggesting bladder cancer until later in the course of the disease. It is important for the differential of a rectal mass to include the possibility of it being a rectal metastasis even if the patient only presents with rectal symptoms initially.

## Introduction

Urothelial carcinoma is a cancer that originates from the urothelial cells that line the renal pelvis, ureters, bladder, and urethra. The bladder is the most common site in the urinary tract where urothelial carcinoma occurs [1]. Urothelial carcinoma is the tenth most common cancer globally [2]. There is a five-year overall survival rate of less than 5% for urothelial carcinoma with metastasis [2]. Bladder cancer usually occurs in individuals older than 55 years and is four times more likely to occur in males than females. Known risk factors for urothelial carcinoma are aging, smoking, arsenic, and exposure to carcinogens [2]. Smoking is the most important risk factor for bladder cancer and accounts for about two-thirds of bladder cancer cases [3,4]. Other common symptoms of bladder cancer include hematuria, urinary incontinence, and dysuria. The standard of care for urothelial carcinoma varies depending on tumor staging, but for urothelial carcinoma with metastasis, it is cisplatin-based chemotherapy [5]. Usually, when urothelial carcinoma metastasizes, it spreads to the bones, lungs, and lymph nodes [6]. However, rectal metastasis from transitional urothelial carcinoma is a very rare occurrence. A rectal metastasis can cause perineal pain and fecal incontinence due to metastasis constricting the rectal lumen and causing obstruction-like symptoms [7]. 

This case report describes an unusual case of high-grade urothelial carcinoma metastasized to the rectum, causing obstruction-like symptoms. Upon review of English literature, about 14 case studies, including this one, have reported transitional urothelial carcinoma metastasizing to the rectum and causing rectal constriction. However, this case is notably remarkable in that the patient, with no previous medical history of cancer, presented with very early symptoms and imaging of rectal malignancy leading to an initial suspicion and workup towards primary colorectal cancer instead of primary urothelial carcinoma. The patient also had a unique presentation of the disease, suffering from bilateral hydronephrosis, fecal incontinence, and a case of large bowel obstruction versus ileus as complications, likely from the malignancy in the colorectum and bladder.

## Case presentation

A 62-year-old Caucasian male, who was formerly a 15 pack-years smoker, presented initially to a general surgeon's clinic with perineal pain, anal discharge, mild difficulty urinating, and constipation. Initially, a digital rectal exam was attempted but was not completed successfully, which prompted a colonoscopy to be scheduled. It was noted that during the attempted digital rectal exam, a rectal mass was appreciated and the rectal lumen was narrow. The first CT pelvis with contrast revealed that the rectum, sigmoid colon, and proximal descending colon had circumferential wall thickening with retroperitoneal enlarged lymph nodes. This prompted the physician's recommendation for a rectal endoscopy with biopsy. It was difficult to pass the scope through the anal canal due to a considerably narrowed lumen. Endoscopic biopsies were taken of the area with easy friability and bleeding. The pathology was inconclusive; thus, both primary colorectal and metastatic lesions were within the differential diagnosis, and additional tissue sampling was recommended.

The first pathology report found the following results: Cytokeratin 7 (CK7) - positive within carcinoma cells in underlying lymphovascular spaces; CK20 - appeared negative within a few remaining neoplastic carcinoma cells; and GATA3 - weak positivity within neoplastic carcinoma cells. The pathologist's report stated that a combination of CK7+/CK20- results would include, but were not limited to, lung, breast, and upper gastrointestinal tract tumors. The weak GATA3 immunoreactivity is thought to be nonspecific, but could also be seen in urothelial and breast origin tumors. CK20+ results could be seen in gastrointestinal tract tumors. 

**Figure 1:**
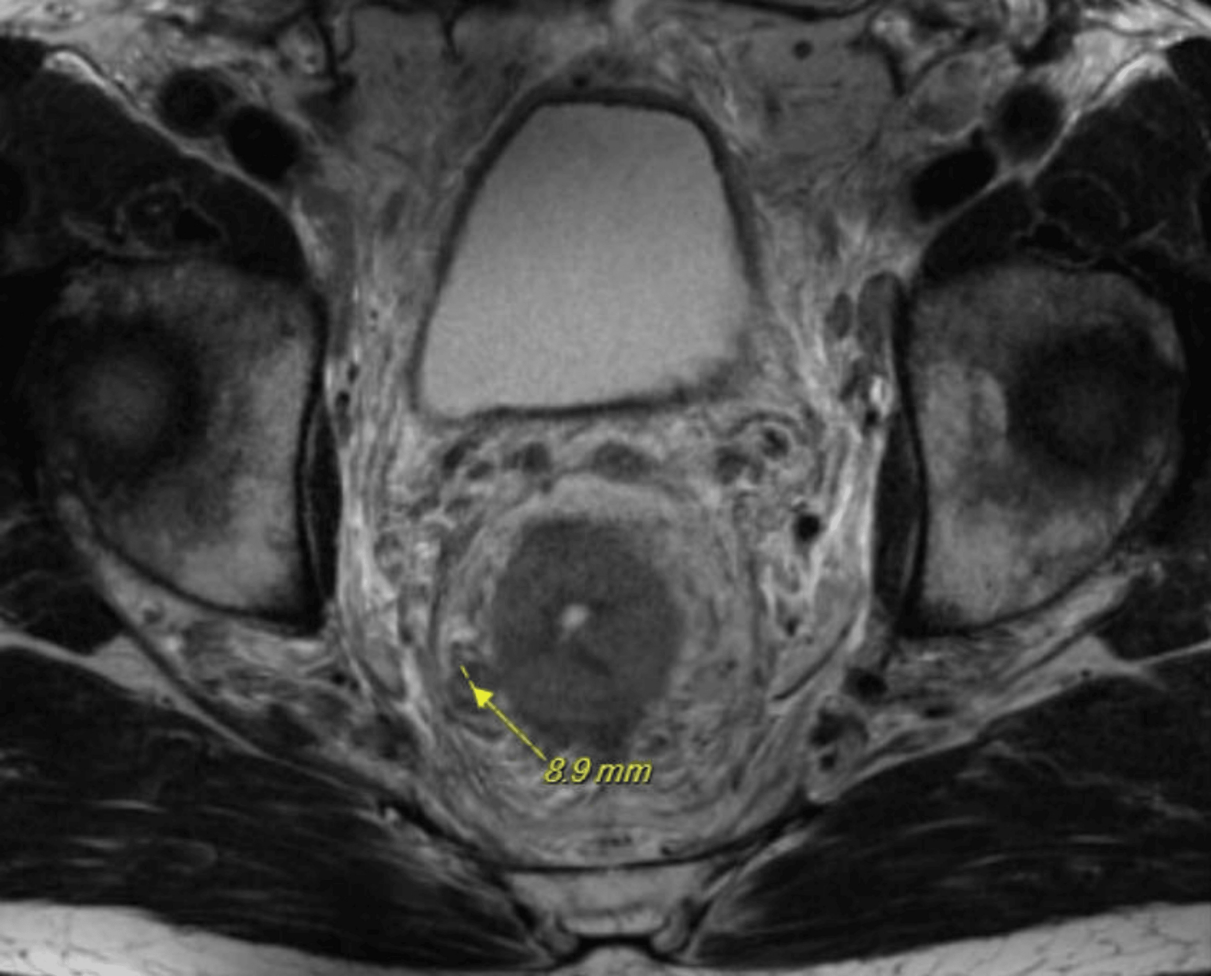
MRI of the pelvis with and without contrast Axial T2-weighted MRI reveals concentric mass-like wall thickening of the rectum and an enlarged mesorectal lymph node measuring 8.9 mm (yellow arrow).

**Figure 2:**
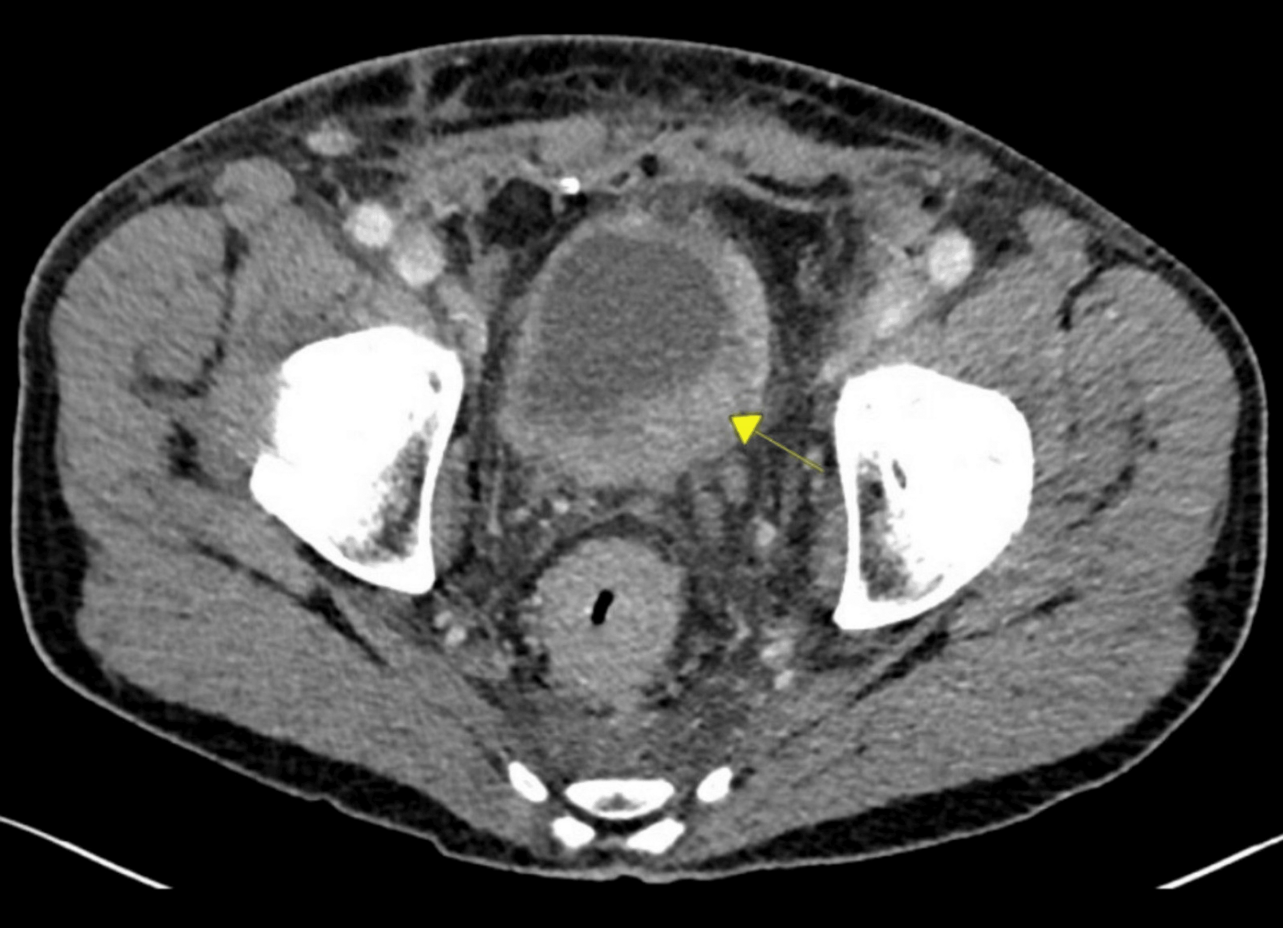
A CT abdomen and pelvis with contrast CT reveals irregular thickening of the bladder concerning for malignancy within the bladder (yellow arrow).

**Figure 3:**
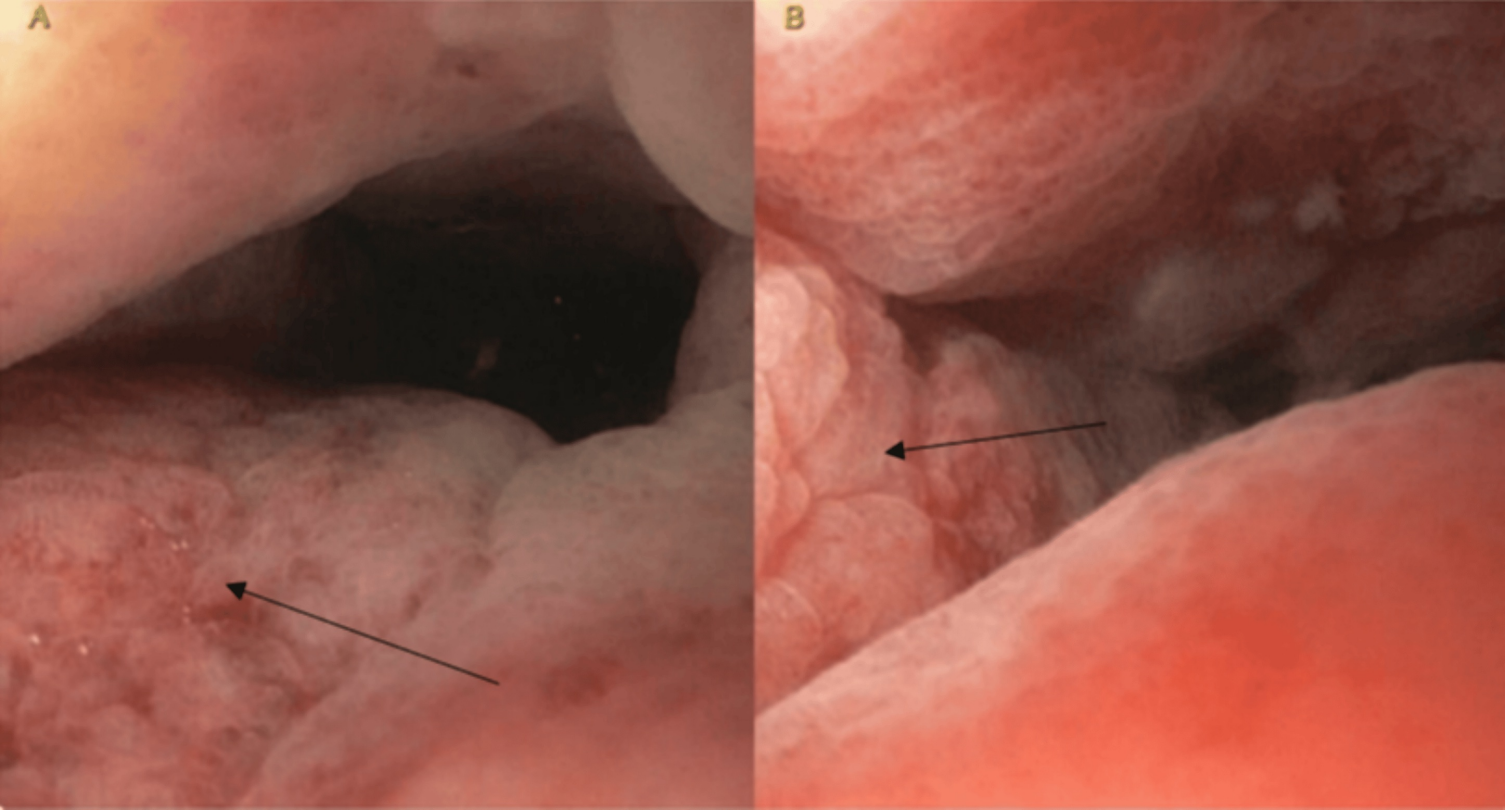
Rectal endoscopic images (A) and (B) The rectal endoscopic images reveal abnormal rectal mucosa (depicted by the black arrows), likely suggestive of invasive malignancy, approximately 13 to 14 cm from the anal verge. In pictures A and B, the abnormal rectal mucosa appears erythematous, thickened, and appears to be folded.

A pelvic MRI with and without contrast revealed marked concentric mass-like wall thickening of the entire rectum within an extent of nearly 12 cm and potentially involving the internal sphincter complex, with extensive mesorectal and pelvic lymphadenopathy (Figure 1). A positron emission tomography (PET) scan was also completed and revealed a hypermetabolic rectosigmoid lesion and hypermetabolic cervical, thoracic, abdominal, and pelvic adenopathy. The patient presented to the emergency department three weeks later due to worsening symptoms. The patient also stated that it was now taking him a long time to initiate urination and he had trouble maintaining a urinary stream, often needing to “step in running water” to be able to urinate. Lab values were obtained, revealing a creatinine level of 1.53 mg/dL and a glomerular filtration rate (GFR) of 50 mL/min/1.73m^2^. A second CT abdomen and pelvis with IV contrast was ordered and revealed left-sided hydronephrosis, and the rectal mass with extensive rectal wall thickening was again seen similar to the prior study. There was new significant mesenteric/omental caking as well as adenopathy in the abdomen and retroperitoneum. Additionally, there was now significant irregular thickening of the bladder wall observed on imaging and concern for a bladder mass was called on this CT (Figure 2). A rectal endoscopic ultrasound (EUS) with biopsies revealed abnormal mucosa suggestive of invasive malignancy extending from the anal verge up to 13 to 14 cm with the previous circumferential involvement of the rectum (Figure 3). The rectal EUS biopsies found the rectal mass to be high-grade metastatic urothelial carcinoma.

The pathology report revealed colorectal mucosa in which there was an underlying high-grade malignancy extending from the submucosa through the muscularis mucosa, focally into the lamina propria with lymphovascular invasion. The carcinoma was CK7, CK5/6, P40, and GATA3 positive, favoring a high-grade urothelial carcinoma.

**Figure 4:**
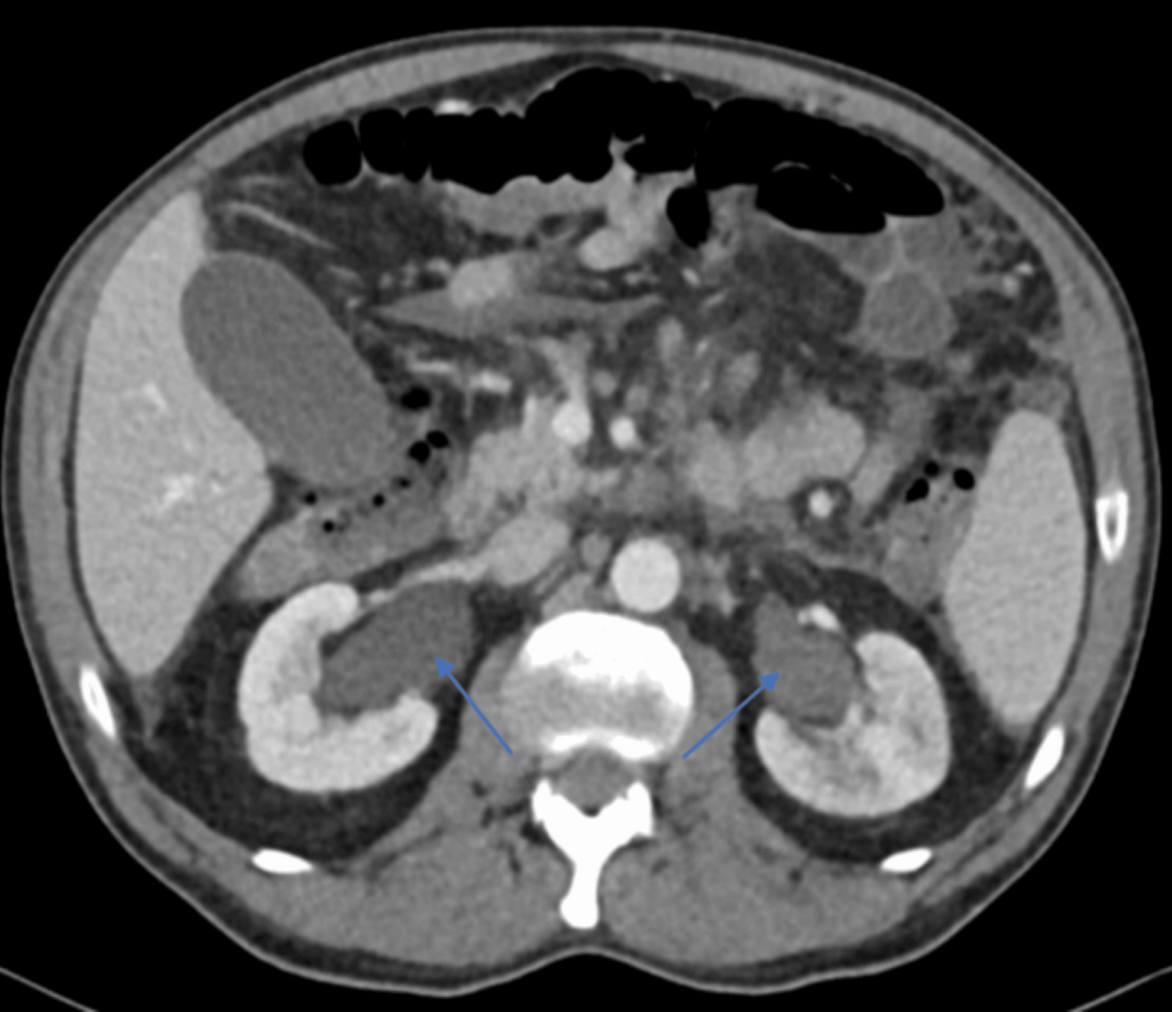
A CT abdomen and pelvis with contrast CT reveals moderate bilateral hydronephrosis (depicted by blue arrows).

About 1.5 months after the patient's initial presentation, his symptoms continued to worsen with increasing difficulty urinating, increased perineal pain to the point where the patient could not ambulate without pain, and difficulty passing bowel movements with occasional loose stools. A third CT abdomen and pelvis with contrast was completed and found moderate bilateral hydroureteronephrosis along with previously reported imaging findings (Figure 4). A cystoscopy and transurethral resection of bladder tumor (TURBT) were completed and found an infiltrative process involving the bladder trigone and bilateral hydroureteronephrosis. The right ureteral orifice was obstructed so a wire and stent could not be passed, while the left ureter had very narrow caliber obstruction for the first approximately 4 cm. An attempt to advance an open-ended catheter over the left wire was met with a significant degree of resistance in the left distal ureter. This led to concern about a malignant infiltrative process of the bladder. The urologist decided that attempts at ureteral dilation should not be done due to the risk of spreading the malignancy and the high likelihood that ureteral stenting would fail to get adequate upper tract drainage. Bilateral nephrostomy tubes were inserted to provide appropriate drainage of his upper urinary tract and reverse the acute kidney injury. Biopsies from the TURBT were obtained from the infiltrative process involving the bladder trigone and pathology reported high-grade urothelial carcinoma invading the muscularis propria. Once the bilateral nephrostomy tubes were placed, the patient’s renal function began to improve, with creatinine decreasing to 1.16 mg/dL and GFR increasing to 70 mL/min/1.73m^2^. Over time, the patient continued to have bowel symptoms of constipation and diarrhea, and began to develop fecal incontinence despite medical management. He continued to have pain in the perineal area, which became uncontrollable even with pain management. Furthermore, the patient's course was complicated by suspected ileus or large bowel obstruction. An X-ray was notable for ileus versus large bowel obstruction as diffuse air-filled loops of bowel were seen throughout the abdomen with some prominence of the cecal region. This was treated with fluids and a nasogastric tube. While it did alleviate some symptoms, further radiographic imaging revealed persistent bowel and colonic distension. The patient was initially planning to start chemotherapy. However, he opted for transition to hospice care. In hospice, the patient’s symptoms continued to progress, with perineal pain that required further up-titration in pain management, and he started to develop incontinent loose stools. The patient passed away in the hospice within a week.

## Discussion

For urothelial carcinoma, it is very rare to observe rectal metastasis, especially in this patient’s specific disease course. Upon review of the medical literature, about 14 cases, including this case, have reported urothelial carcinoma metastasizing to the rectum and causing rectal constriction. This case is unique considering the rarity of rectal metastasis from urothelial carcinoma and the course of this patient’s disease. It was noteworthy that the rectal symptoms preceded the major urological symptoms, as the patient’s primary presenting symptoms were perineal pain, constipation, and a rectal mass. Initial imaging first found significant findings of colorectal circumferential wall thickening. It should be noted that at presentation, the patient denied symptoms of hematuria and an increased urinary frequency but did complain of mild difficulty urinating. The patient’s primary presenting symptoms and initial image findings led to concern and workup for primary colorectal cancer instead of primary urothelial cancer. Subsequent imaging revealed significant image findings of a bladder mass that was noted by the radiologist. It should be noted that on review of the initial CT image, there was possible evidence of bladder pathology. However, findings were equivocal and may have been artifactual due to a nondistended bladder. Findings of a bladder mass were not specific until the second CT pelvis. Throughout the patient’s overall disease course, rectal constriction from the rectal metastasis likely causing structural constipation and beginning of fecal incontinence likely due to metastasis were observed. Also, during the second rectal endoscopy, it was noted by the gastroenterologist that the internal anal sphincter could be involved with this invasive disease, which may have additionally caused the fecal incontinence. Another finding that makes this case significant is the bilateral hydronephrosis likely caused by the infiltrative process involving the trigone, the right ureteral orifice being obstructed, and the left ureter having a very narrow caliber obstruction on cystoscopy. 

Similar to this case, from a review of the medical literature, the cases that have transitional urothelial carcinoma causing rectal constriction have a common presentation of male biological sex and high tumor grade/stage [8-12]. This case report further supports this observation. Similar studies with urothelial carcinoma metastasizing to the rectum were reported by Takeuchi et al. and Kassam et al. [7,9]. Our patient had symptoms of bilateral hydronephrosis and rectal obstruction similar to a case by Takeuchi et al. [7]. Kassam et al. reported symptoms of large bowel obstruction, comparable to our patient's case of large bowel obstruction vs ileus [9]. A case report by Stillwell, Rife, and Lieber reported two cases of urothelial carcinoma metastasizing to the rectum and both cases reported rectal obstruction [10]. One case in the report had bilateral hydronephrosis. Both cases had a prior diagnosis of bladder cancer and presented later to clinic due to rectal metastasis rather than initially presenting with primary rectal symptoms. Kobayashi et al. reported three cases of primary urothelial carcinoma with rectal metastasis, all of which caused rectal obstruction [11]. Two of these cases reported bilateral hydronephrosis. All three cases found thickened bilateral lateral pedicles and cancer encroached on both ureteral orifices. However, cystoscopy in our study found an infiltrative process on the bladder trigone with bulging at both ureteral orifices and no thickened lateral pedicles. Cases one and three in the study by Kobayashi et al. had patients with previously diagnosed bladder cancer who later presented with rectal metastasis and corresponding clinical manifestations [11]. The patient in Case two initially presented with bladder irritability and perineal pain. The initial CT in Case three called a thickened bladder wall and rectal wall which were similar findings compared to our study. A study by Langenstroer et al. presented a similar case, with the symptom of fecal incontinence throughout the disease course [12]. However, compared to our patient, the patient in the study by Langenstroer et al. did not have bilateral hydronephrosis and the patient had a known diagnosis of urothelial cancer [12]. 

Upon review of medical literature, common symptoms like those in our case include bilateral hydronephrosis, rectal obstruction, male patients, and high-grade urothelial carcinoma. However, unlike most cases in literature, our case initially presents with the clinical manifestations of rectal metastasis with undiagnosed primary urothelial carcinoma. It is unique as it presents additional information to the medical literature due to the combined presentation of how rarely urothelial carcinoma metastasizes to the rectum, how early the rectal metastasis caused symptoms, and the overall disease course, especially how the patient developed all three symptoms of fecal incontinence, bilateral hydronephrosis, and ileus versus a large bowel obstruction. These symptoms likely originated from the progression of the cancer in the bladder and rectum. This case study will be able to provide additional clinical information such as clinical course and symptoms on rectal metastasis from high-grade urothelial carcinoma.

## Conclusions

This case report describes a rare case of high-grade transitional urothelial carcinoma metastasizing to the rectum. The significance of this case is that the initial imaging and symptoms led to concern for colorectal malignancy. However, the patient’s early symptoms of difficulty defecating, a rectal mass, and some difficulty urinating were likely due to rectal metastatic disease, with the major urinary symptoms suggesting bladder cancer emerging later in the patient’s disease course. The patient's primary malignancy and metastasis likely contributed to the bilateral hydronephrosis, fecal incontinence, and a case of large bowel obstruction vs ileus. 

Consequently, metastatic disease should be in the differential for circumferential rectal masses, especially in patients with risk factors for cancer. This is important even in atypical cases since primary colorectal cancer and metastasis appear similar on medical imaging and may present with equivocal symptoms. Based on the physician’s recommendations, a tissue biopsy of the rectal mass was essential to further guide clinical workup and was a necessary step to correctly diagnose the cancer. The standard of care for urothelial carcinoma is chemotherapy. However, upon patient-provider discussion, the patient ultimately decided to enter hospice care which better aligned with their goals of care. This case report will provide further context for past and future studies on how urothelial carcinoma can metastasize and cause rectal constriction and the different types of symptoms it can cause.
